# Expansion of the spectrum of tumors diagnosed as myxopapillary ependymomas

**DOI:** 10.1007/s00401-025-02944-w

**Published:** 2025-09-30

**Authors:** Fuat Kaan Aras, Dennis Friedel, Felix Keller, Ferdinand Zettl, Rouzbeh Banan, Philipp Sievers, Abigail K. Suwala, Felix Hinz, Lukas Friedrich, Ivan Abdulrazak, Mozhgan Esmaeilibenvidi, Nima Etminan, Christel Herold-Mende, Wolfgang Wick, Sandro Krieg, Stefan M. Pfister, Andrey Korshunov, Isabell Bludau, Felix Sahm, David E. Reuss, Gianluca Sigismondo, Andreas von Deimling

**Affiliations:** 1 Department of Neuropathology, Institute of Pathology, University Hospital Heidelberg, 69120 Heidelberg, Germany; 2 Clinical Cooperation Unit Neuropathology, German Consortium for Translational Cancer Research (DKTK), German Cancer Research Center (DKFZ), Heidelberg, Germany; 3 Department of Neurosurgery, University Hospital Mannheim, University of Heidelberg, Mannheim, Germany; 4 Division of Experimental Neurosurgery, Department of Neurosurgery, University Hospital Heidelberg, Heidelberg, Germany; 5 Department of Neurology, Heidelberg University Hospital, Heidelberg, Germany; 6 a Partnership Between DKFZ and Heidelberg University Hospital, National Center for Tumor Diseases (NCT), NCT Heidelberg, Heidelberg, Germany; 7 Clinical Cooperation Unit Neurooncology, German Consortium for Translational Cancer Research (DKTK), German Cancer Research Center (DKFZ), Heidelberg, Germany; 8 Department of Neurosurgery, Heidelberg University Hospital, Heidelberg, Germany; 9 Hopp Children’s Cancer Center Heidelberg (KiTZ), Heidelberg, Germany; 10 Department of Pediatric Oncology, Hematology, Immunology and Pulmonology, University Hospital Heidelberg, Heidelberg, Germany; 11 Faculty of Bioscience, Heidelberg University, 69120 Heidelberg, Germany

Both spinal ependymomas (SPE) and myxopapillary ependymomas (MPE) exhibit a characteristic methylation profile [6]. However, for a significant portion of SPE, many pathologists have reported conflicting results between morphologic diagnosis and the DNA methylation-based classification [9, 11]. Indeed, a study by the German Glioma Network reported that nearly one-third of the histologically diagnosed spinal ependymomas were assigned by methylation to the MPE class [14]. In the current study, we address this topic and focus on SPE cases exhibiting a methylation profile of MPE.

We performed immunohistochemical, AI-assisted morphological, as well as methylation analyses on a cohort of 100 MPE and SPE. In addition, mass spectrometry-based proteomic analysis was conducted on 54 of these tumors, including 16 MPE, 23 SPE and 15 discrepant cases. A detailed description of methods is provided in supplementary materials. Data refinement included filtering for precursor peptides and proteins with q-values > 0.01. Next, low-quality samples with less than 5000 identified proteins were excluded. Additionally, proteins with a high incidence of missing values (i.e., more than 50% in both MPE and SPE) were excluded from the analysis. This approach removed 10 tumor samples, with the final cohort constituting 16 MPE, 17 SPE, and 11 discrepant cases. In these samples, we identified by MS/MS an average of 69,658 (± 12,648) peptides and 8029 (± 758) proteins per sample at a false discovery rate (FDR) lower than 1% at both peptide and protein level.

Pearson correlation matrix showed higher similarity between discrepant cases and MPEs, compared to discrepant cases and SPEs (Fig. 1). In agreement, principal component analysis (PCA) revealed weakly predominant clustering of the discrepant cases with MPEs (Fig. 1). In addition, proteomic analysis identified HOXB13 as the most differentially expressed protein between MPE and SPE. HOXB13 was indeed highly prevalent in all MPEs (16 out of 16 samples) and not identified in any of the SPEs (0 out of 17 samples) (Supplementary Fig. 1). Immunohistochemical staining in a cohort of 100 tumor samples demonstrated 100% sensitivity and 100% specificity (Supplementary Figs. 2 and 3). Differently from previous reports, we did not observe weak or focal staining of HOXB13 in the tumor samples [2, 8, 11, 13]. This can be attributed to the usage of a different antibody compared to previous research (Supplementary Materials). HOXB13 showed strong and diffuse positive staining in all discrepant cases, thus perfectly aligning with proteomic data.

**Fig. 1:**
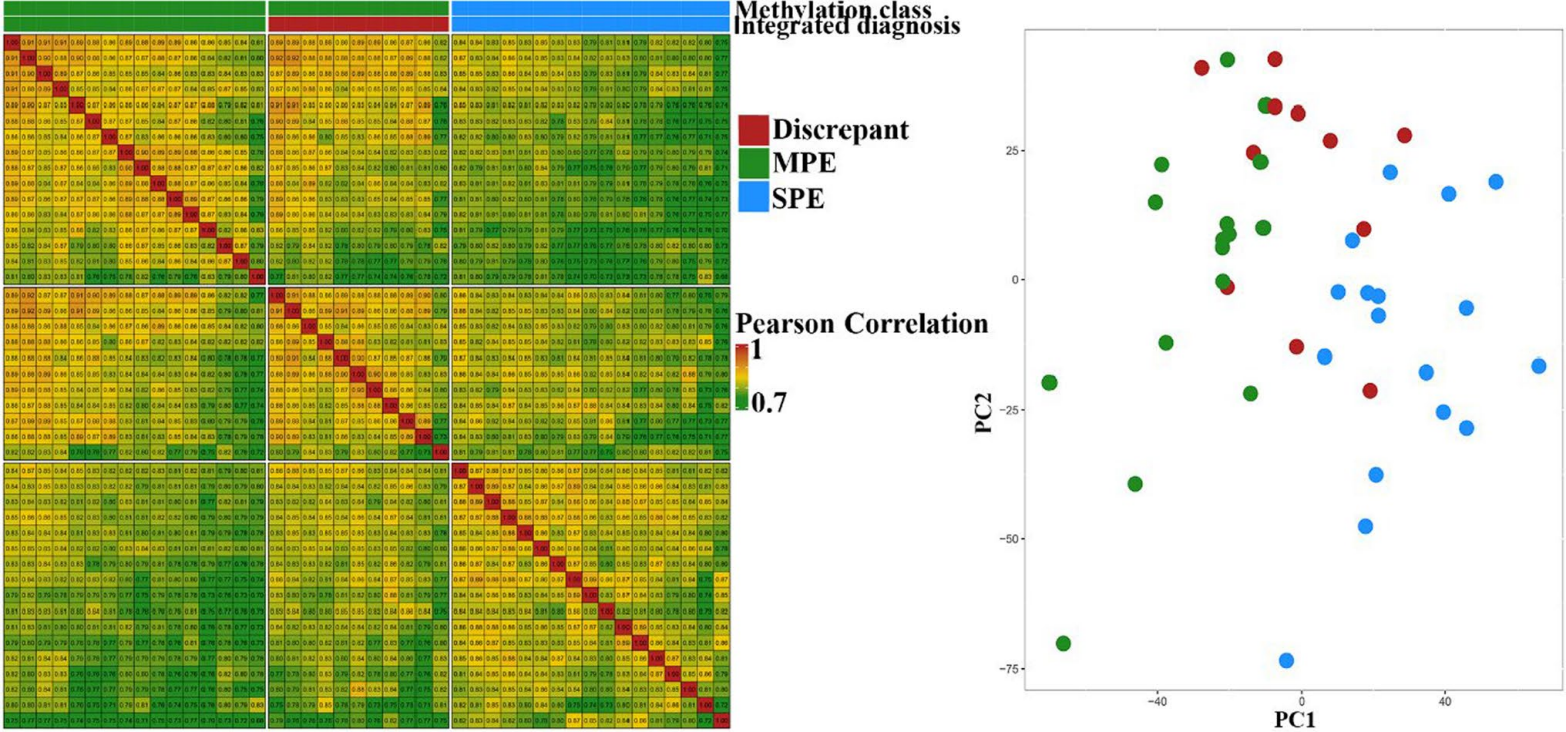
Pearson correlation matrix (left panel) and principal component analysis (right panel) of proteomes of 44 tumor samples

Next, we investigated the morphological features of the cohort. The extracellular proteoglycan versican (VCAN) was overrepresented in the myxoid matrix of MPE (Supplementary Fig. 1). VCAN immunohistochemistry showed excellent alignment with Alcian blue stain; however, VCAN immunohistochemistry demonstrated a more prominent and reliable staining. Employing digital pathology tools and VCAN immunohistochemistry, we demonstrated that discrepant cases frequently contain myxoid *foci* (Supplementary Figs. 4–6 and Supplementary Table 1).

CNV summary plots of MPEs and SPEs were compatible with previous reports [7]. Noteworthy, discrepant cases appeared to exhibit a lower rate of chromosomal aberrations in comparison to MPEs and SPEs. Overall, the summary CNV plot of discrepant cases was more similar to MPE than SPE (Supplementary Fig. 7).

The morphologic and methylation-based classification difference between MPE and SPE has been the subject of several studies [9, 11, 14]. Also, the observation of strong expression of HOXB13 in a fraction of ependymomas with a focus on MPE has been previously reported [2, 3, 5, 8, 11, 13]; thus, our observations corroborate previous reports. As a unique feature, the present study focuses on the conflicting morphological and methylation-based classification of MPE and SPE, while proposing a systematic approach to resolve this discrepancy.

Previous studies observed a differential expression of HOXB13 in morphologically diagnosed MPE [2, 13]. A subsequent study demonstrated strong HOXB13 expression predominantly in MPE, but not in other spinal ependymomas or astrocytomas [8]. After the advent of methylation-based classification, a considerable fraction of morphologically unequivocal SPE produced an mcMPE profile, as reported in several studies [9, 10, 14].

Altogether, the different layers of information demonstrated that discrepant cases show a pattern more similar to MPE than SPE. Our results speak in favor of extending the spectrum of tumors diagnosed as MPE. The diagnosis of an MPE should include the discrepant cases with an SPE-like morphology, but with nuclear expression of HOXB13, and a methylation profile of myxopapillary ependymoma. Adoption of such a pipeline in clinical practice would require modifications of the WHO definition and the essential and desirable diagnostic criteria for these tumors.

It should be noted that cauda equina neuroendocrine tumor, a tumor within the extended differential diagnosis of MPE and SPE, also exhibits nuclear expression of HOXB13 [1, 4, 12].

We have not observed a classical MPE without strong nuclear HOXB13 expression. We therefore propose classifying tumors that resemble morphologically SPE, but exhibit an mcMPE profile or have nuclear expression of HOXB13 as MPE irrespective of their morphological appearance. This would imply considerable changes to the criteria provided by WHO for the diagnosis of MPE. A suggestion is presented in Fig. 2.

**Fig. 2:**
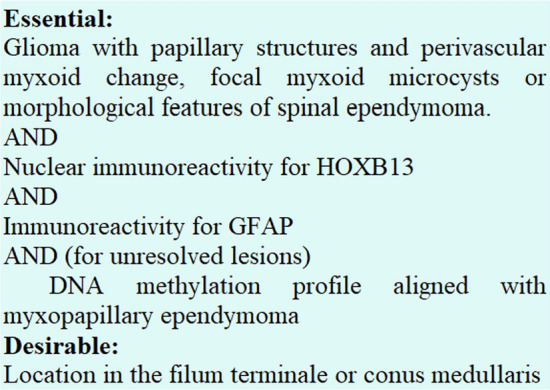
Proposal for diagnostic criteria of myxopapillary ependymoma

## Supplementary Information

Below is the link to the electronic supplementary material.

Supplementary file1 (XLSX 19 KB)

Supplementary file2 (DOCX 7630 KB)

## Data Availability

Data are deposited into a public repository for mass spectrometry data (PRIDE, https://www.ebi.ac.uk/pride/) and the credentials for access will be shared with the reviewers during revision. Data will be publicly available upon successful acceptance.

## References

[1] Asa SL, Mete O, Schüller U et al (2022) Cauda equina neuroendocrine tumors. Am J Surg Pathol 47:469–475. 10.1097/pas.0000000000002009 36543154 10.1097/PAS.0000000000002009

[2] Barton VN, Donson AM, Kleinschmidt-DeMasters BK et al (2009) Unique molecular characteristics of Pediatric myxopapillary ependymoma. Brain Pathol 20:560–570. 10.1111/j.1750-3639.2009.00333.x 19793339 10.1111/j.1750-3639.2009.00333.x PMC2871180

[3] Bockmayr M, Harnisch K, Pohl LC et al (2022) Comprehensive profiling of myxopapillary ependymomas identifies a distinct molecular subtype with relapsing disease. Neuro-Oncol 24:1689–1699. 10.1093/neuonc/noac088 35380708 10.1093/neuonc/noac088 PMC9527524

[4] Bockmayr M, Körner M, Schweizer L, Schüller U (2021) Cauda equina paragangliomas express HOXB13. Neuropathol Appl Neurobiol 47:889–890. 10.1111/nan.12713 33768604 10.1111/nan.12713

[5] Bschorer M, Dottermusch M, Matschke J et al (2024) 40-year-old man with two asynchronous spinal cord tumors. Brain Pathol. 10.1111/bpa.13309 39370146 10.1111/bpa.13309 PMC11483506

[6] Capper D, Jones DTW, Sill M et al (2018) DNA methylation-based classification of central nervous system tumours. Nature 555:469–474. 10.1038/nature26000 29539639 10.1038/nature26000 PMC6093218

[7] Capper D, Stichel D, Sahm F et al (2018) Practical implementation of DNA methylation and copy-number-based CNS tumor diagnostics: the Heidelberg experience. Acta Neuropathol 136:181–210. 10.1007/s00401-018-1879-y 29967940 10.1007/s00401-018-1879-y PMC6060790

[8] Gu S, Gu W, Shou J et al (2015) The molecular feature of HOX gene family in the intramedullary spinal tumors. Spine 42:291–297. 10.1097/brs.0000000000000889 10.1097/BRS.0000000000000889 25785959

[9] Kresbach C, Neyazi S, Schüller U (2022) Updates in the classification of ependymal neoplasms: the 2021 WHO classification and beyond. Brain Pathol. 10.1111/bpa.13068 35307892 10.1111/bpa.13068 PMC9245931

[10] Pajtler KW, Witt H, Sill M et al (2015) Molecular classification of ependymal tumors across all CNS compartments, histopathological grades, and age groups. Cancer Cell 27:728–743. 10.1016/j.ccell.2015.04.002 25965575 10.1016/j.ccell.2015.04.002 PMC4712639

[11] Purkait S, Praeger S, Felsberg J et al (2025) Strong nuclear expression of HOXB13 is a reliable surrogate marker for DNA methylome profiling to distinguish myxopapillary ependymoma from spinal ependymoma. Acta Neuropathol. 10.1007/s00401-025-02866-7 40137996 10.1007/s00401-025-02866-7 PMC11947044

[12] Soukup J, Manethova M, Stejskal V et al (2023) Immunoreactivity of HOXB13 in neuroendocrine neoplasms is a sensitive and specific marker of rectal well-differentiated neuroendocrine tumors. Endocr Pathol 34:333–341. 10.1007/s12022-023-09779-9 37552455 10.1007/s12022-023-09779-9

[13] Stephen JH, Sievert AJ, Madsen PJ et al (2012) Spinal cord ependymomas and myxopapillary ependymomas in the first 2 decades of life: a clinicopathological and immunohistochemical characterization of 19 cases. J Neurosurg Pediatr 9:646–653. 10.3171/2012.2.peds11285 22656257 10.3171/2012.2.PEDS11285

[14] Witt H, Gramatzki D, Hentschel B et al (2018) DNA methylation-based classification of ependymomas in adulthood: implications for diagnosis and treatment. Neuro-Oncol 20:1616–1624. 10.1093/neuonc/noy118 30053291 10.1093/neuonc/noy118 PMC6231197

