# Supplementary material for: Expansion of the spectrum of tumors diagnosed as myxopapillary ependymomas
Source: Acta Neuropathol. 2025 Sep 30;150(1):37. doi: 10.1007/s00401-025-02944-w (PMC12484246; doi:10.1007/s00401-025-02944-w)
Supplement: Supplementary file 2 — Supplementary file2 (DOCX 7630 KB) [file 401_2025_2944_MOESM2_ESM.docx]

**SUPPLEMENTARY MATERIALS**

***Acta Neuropathologica***

**Expansion of the spectrum of tumors diagnosed as myxopapillary ependymomas**

Fuat Kaan Aras^1,2^ Dennis Friedel^1,11^ Felix Keller^1,2^ Ferdinand Zettl^1,2^ Rouzbeh Banan^1,2^ Ivan Abdulrazak^1,2^ Mozhgan Esmaeilibenvidi^1,2^ Nima Etminan^3^ Christel Herold-Mende^4^ Wolfgang Wick^5,6,7^ Sandro Krieg^8^ Stefan M. Pfister^9,10^ Andrey Korshunov^1,2^ Isabell Bludau^1,2^ Felix Sahm^1,2^ David E. Reuss^1,2^ Gianluca Sigismondo^1,2^ Andreas von Deimling^*1,2^

^1^ Department of Neuropathology, Institute of Pathology, University Hospital Heidelberg, 69120 Heidelberg, Germany

^2^ Clinical Cooperation Unit Neuropathology, German Consortium for Translational Cancer Research (DKTK), German Cancer Research Center (DKFZ), Heidelberg, Germany

^3^ Department of Neurosurgery, University Hospital Mannheim, University of Heidelberg, Mannheim, Germany

^4^ Division of Experimental Neurosurgery, Department of Neurosurgery, University Hospital Heidelberg, Heidelberg, Germany

^5^ Department of Neurology, Heidelberg University Hospital, Heidelberg, Germany

^6^ National Center for Tumor Diseases (NCT), NCT Heidelberg, a Partnership Between DKFZ and Heidelberg University Hospital, Heidelberg, Germany

^7^ Clinical Cooperation Unit Neurooncology, German Consortium for Translational Cancer Research (DKTK), German Cancer Research Center (DKFZ), Heidelberg, Germany

^8^ Department of Neurosurgery, Heidelberg University Hospital, Heidelberg, Germany

^9^ Hopp Children's Cancer Center Heidelberg (KiTZ), Heidelberg, Germany

^10^ Department of Pediatric Oncology, Hematology, Immunology and Pulmonology, University Hospital Heidelberg, Heidelberg, Germany

^11^ Faculty of Bioscience, Heidelberg University, 69120 Heidelberg, Germany

**Corresponding author:**

^*^Andreas von Deimling, MD

Department of Neuropathology, Institute of Pathology, University Hospital Heidelberg

Heidelberg, Germany

andreas.vondeimling@med.uni-heidelberg.de

**TABLE OF CONTENTS**

**Methods……………………………………………………………………………………..3-5**

**Supplementary Figure 1……………………………………………………………………...5**

**Supplementary Figure 2……………………………………………………………………...6**

**Supplementary Figure 3……………………………………………………………………...6**

**Supplementary Figure 4……………………………………………………………………...7**

**Supplementary Figure 5……………………………………………………………………...7**

**Supplementary Figure 6……………………………………………………………………...8**

**Supplementary Figure 7……………………………………………………………………...9**

**References……………………………………………………………………………………..9**

**Case selection**

The study cohort consisted of spinal and myxopapillary ependymomas with a score higher than 0.9 score in their assigned DNA methylation class according to the Heidelberg brain tumor classifier v12b8. 121 cases were found to fill these criteria. A case was defined as discrepant if its histological diagnosis is spinal ependymoma (SPE) and its Heidelberg methylation classifier v12b8 prediction is myxopapillary ependymoma (MPE). Determination of the histological diagnosis was based on the neuropathological reports retrospectively in order to avoid bias. 4 cases were excluded due to uncertainty of their discrepancy status. 100 cases with available formalin-fixed paraffin-embedded (FFPE) material were collected from the archives of the departments of neuropathology and neurosurgery at the Heidelberg and Mannheim university hospitals. 54 cases which contain sufficient and high-quality material in their FFPE blocks were used for further proteomic analyses. Tissue and data collection were performed in consideration of local ethics regulations and approval. A summary of our cohort is provided in Supplementary Table 1. All cases of the proteomic evaluation cohort were evaluated and marked for tissue extraction by at least two neuropathologists using HE-stained tumor sections.

**Methylation analysis and summary copy number variation plots**

Genome-wide DNA methylation analysis was conducted using the Infinium Methylation 450k and Illumina MethylationEPIC (EPIC) BeadChip (Illumina, San Diego, USA), as previously described [1], and in accordance with the manufacturer’s protocols. Data analysis was performed using R version 4.6.1.. All samples underwent individual background and colour channel dye-bias correction for normalisation.. Background correction was achieved by shifting the 5th percentile of the negative control CpG to 0. For dye-bias correction, both colour channels had their normalisation control CpG intensities scaled to 10,000. Subsequently, a batch correction between array types (450k/EPIC) was performed on the log2-transformed intensity values using the *removeBatchEffect* function from the R-package limma (version 3.30.11) [2]. Retransformed intensities were employed to derive beta-values, with an offset of 100, as per the recommendations provided by Illumina.

Copy number variation (CNV) analysis was conducted for each array type for each sample by using the R-package “conumee”. Summary CNV plots were generated by segmenting the chromosomal arms into bins of 1%, thereby enabling the estimation of the relative frequency of gains and losses within each group. These values were subsequently plotted alongside a genomic axis that spans from 1 to 22 using the plot function in R, with X representing the 1% of the chromosomal arms and Y indicating the percentage of relative frequency of gains and losses.

**Sample preparation of FFPE tissue for mass spectrometry**

For proteomic analysis, 1.5 mm in diameter biopsy punches of FFPE blocks were subjected to protein extraction protocol with the BeatBox device (Preomics, Planegg/Martinsried, Germany). In brief, four cycles of BeatBox-mediated tissue shearing in SDS 4% lysis buffer were performed, followed by SP3-based protein clean-up [3], and on-bead tryptic digestion (Promega, Fitchburg, WI, USA) in 20uL of 50mM ammonium bicarbonate at 37 Celcius degrees for 16 hours, 800 rpm. Resulting peptides were acidified to TFA 1% final, and stored at -20 celcius degrees until the mass spectrometry injection.

**LC-MS/MS acquisition by DIA-PASEF mode**

A total of 200ng of peptides were separated on a 25 cm analytical column (75μm x 250mm, C18, 1.7μm, 120 Å, Aurora 3, IonOpticks) using the nanoElute2 liquid chromatography system (Bruker Daltonics, Billerica, MA, USA) coupled to a Trapped Ion Mobility Spectrometry Time-of-Flight HT Mass Spectrometer (Bruker Daltonics, Billerica, MA, USA) operated in positive mode (+1.6kV). Solvent A was water with 0.1% formic acid and solvent B was 80% acetonitrile, 0.1% formic acid. Peptides were separated over 120min at 50C, the percentage of solvent B increased in a linear fashion from 3% to 8% in 3 min, then increased to 10% at 15 min, to 40% at 100 min, to 60% in 105 min and finally to 90% at 110 min, stayed 5min at 90%, and went down to 3% for the last 5 min. Peptides were acquired in data-independent acquisition mode with parallel accumulation - serial fragmentation (dia-PASEF), in a 100 – 2000 m/z range, and a 0.65 – 1.4 1/k0 ion mobility space, with a total cycle time of 1s.

**Whole proteome analysis**

Identification and quantification of peptides and proteins from raw spectra data was conducted via the proteomic analysis tool DIA-NN (version 1.9) [4] by using default settings specifically described for TIMS-TOF instruments. Subsequent statistical analysis of whole proteome data was carried out in the programming language R version 4.3.1.. Prior imputation, protein intensities were normalised using the median centering normalisation implementation from the R-package “proBatch” [5]. In the following, remaining missing values were imputed by using minDet imputation from the R-package “imputeLCMD”. Similarity analysis between the whole proteome samples was carried out by calculating the Pearson correlation between the protein expression profiles of the cases. The correlations were then visualized as heatmap using the R-package Complex Heatmap [6]. A principal component analysis was conducted on the pre-processed and imputed protein intensities to investigate similarities in expression programs of the analysed patient samples. The sample distribution in the first two principal components were used for graphical interrogation and visualized by using the R-package „ggplot“. Differential expression analysis was conducted via the R-package “limma” [2] and the Benjamini–Hochberg procedure for FDR was applied for controlling of the resulting p-values [7]. Proteins of interest were defined when the absolute log-fold change exceeded 0.5 and the adjusted P-value was less than 0.05. Volcano plots were generated by using the R-package “enhancedVolcano”. Additional graphical representations if not stated otherwise were generated using “ggplot2” and “patchwork”.

**Digital morphological analyses**

H&E, Alcian blue and VCAN whole slide images were used as input. Computer Vision (CV) methods were also used to highlight areas of RGB values within the expected spectrum of positive colors for Alcian blue stain.

**Immunohistochemistry**

Immunohistochemistry was conducted on 5-µm-thick formalin-fixed, paraffin-embedded (FFPE) tissue sections mounted on StarFrost Advanced Adhesive slides (Engelbrecht, Kassel, Germany). Immunohistochemistry was performed on a BenchMark Ultra immunostainer (Ventana Medical Systems, Tucson, AZ, USA). HOXB13 (#90944, Cell Signaling Technology, Danvers, MA, USA, dilution 1:500), and VCAN (ab270445, Abcam, Cambridge, UK, dilution 1:5000).

Please note that adaptations in antibody concentration and/or pretreatment time to the local technique and parameters like fixation time and age of the blocks may be necessary.

**HOXB13:**

Procedure: U OptiView DAB IHC v6 (v1.00.0136)

BenchMark ULTRA IHC/ISH

1. Increase slide temperature from 72 ° C to medium temperatures (dewaxing).
2. Warm the slide to 100 °C and incubate for 64 minutes (Cell Conditioner No.1).
3. Warm slides to 37 °C.
4. Apply 1 drop of antibody (1:500), apply LCS and incubate for 1 hour.
5. Apply 1 drop of OptiView HQ Linker and incubate in for 8 min.
6. Apply 1 drop of OptiView HRP Multimer and incubate for 8 min.
7. Apply 1 drop of OptiView Amplification H2O2 and OptiView Amplifier, apply LCS and incubate for 4 min.
8. Apply 1 drop OptiView Amplification Multimer and incubate for 4 min.
9. Apply 1 drop of hematoxylin (counterstaining), apply LCS and incubate for 8 minutes.
10. Apply 1 drop of bluing reagent (counterstaining), apply LCS and incubate for 4 minutes.

**VCAN:**

Procedure: U UltraView DAB (v1.02.0018)

BenchMark ULTRA IHC/ISH

1. Increase slide temperature from 72 ° C to medium temperatures (dewaxing)
2. Warm slides to 95 ° C and incubate for 56 minutes (Cell Conditioner No. 2).
3. Warm slides to 37 ° C and incubate for 4 minutes.
4. Apply 1 drop of antibody (1:5000 dilution), apply LCS and incubate for 32 min.
5. Apply 1 drop of hematoxylin (counterstaining), apply LCS and incubate for 8 minutes.
6. Apply 1 drop of bluing reagent (counterstaining), apply LCS and incubate for 4 minutes.


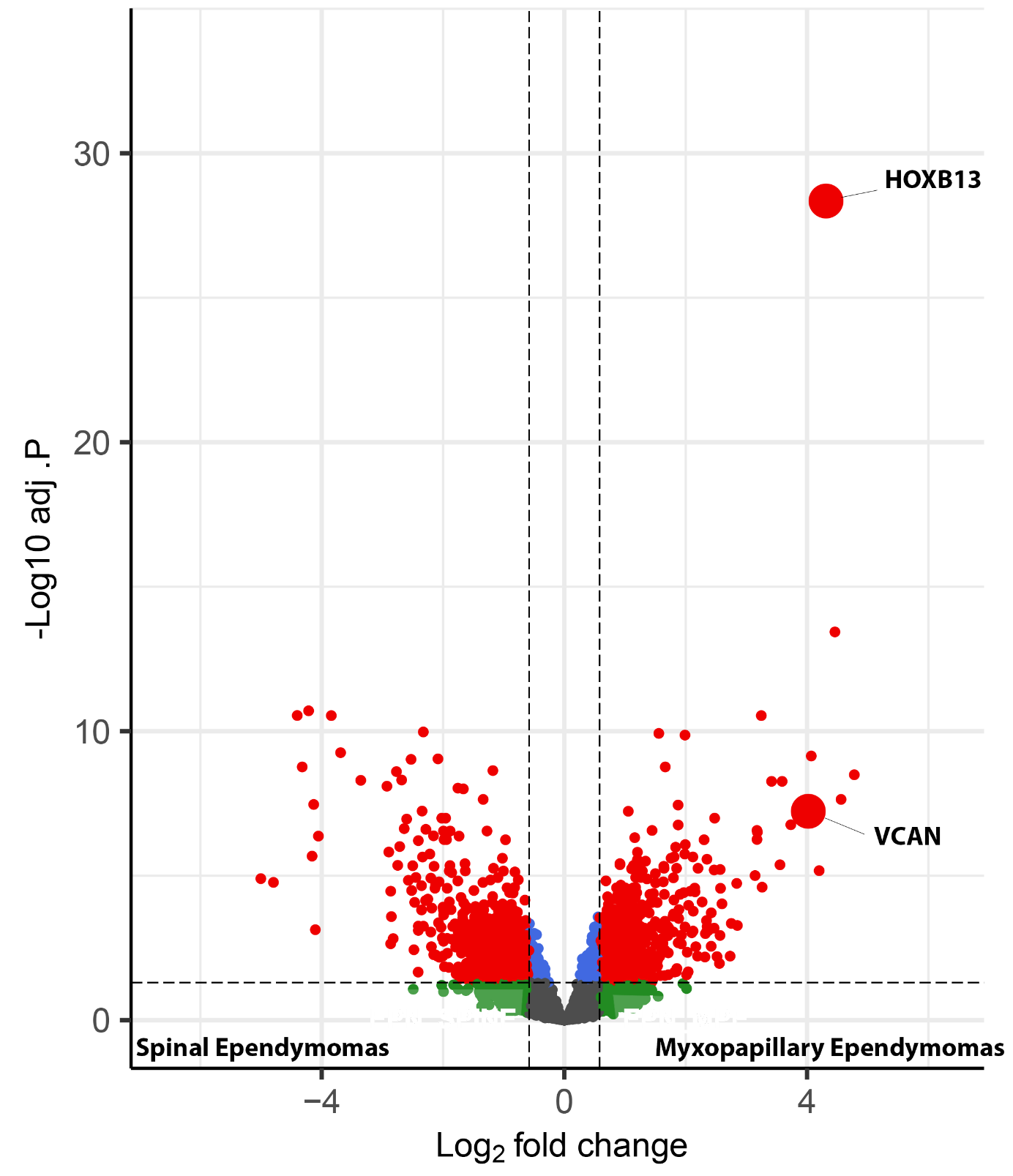
 **Suppl Fig. 1** Volcano plot of differentially expressed proteins between spinal and myxopapillary ependymomas including discrepant cases as myxopapillary ependymomas.


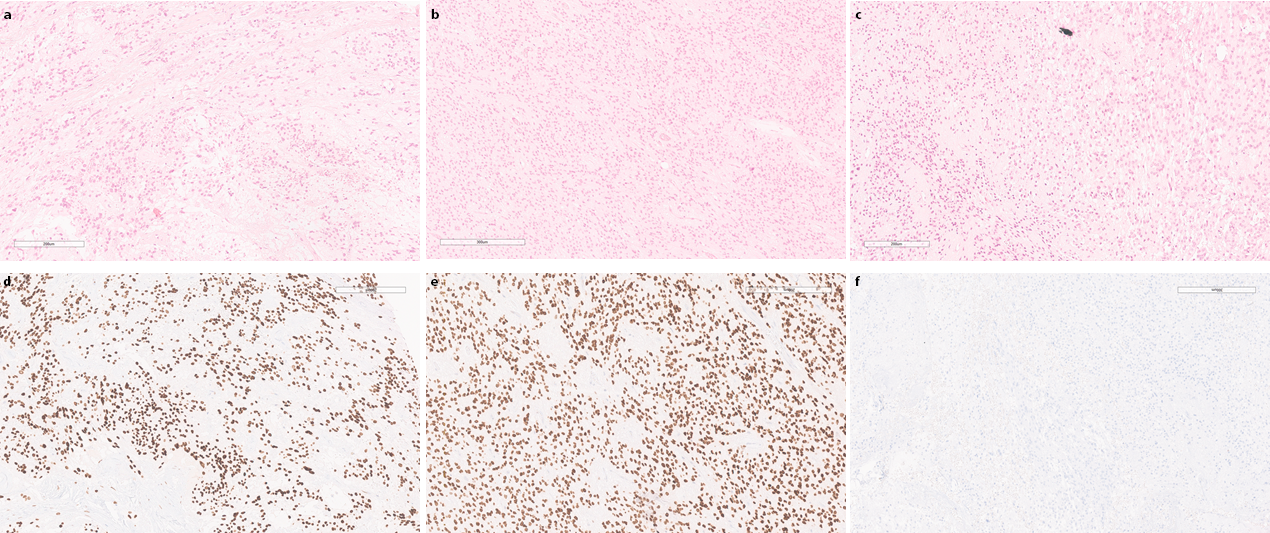


**Suppl Fig. 2** Pairwise comparison of HE slides and corresponding HOXB13 immunohistochemistry of myxopapillary ependymomas (a and d), discrepant cases (b and e) and spinal ependymomas (c and f).


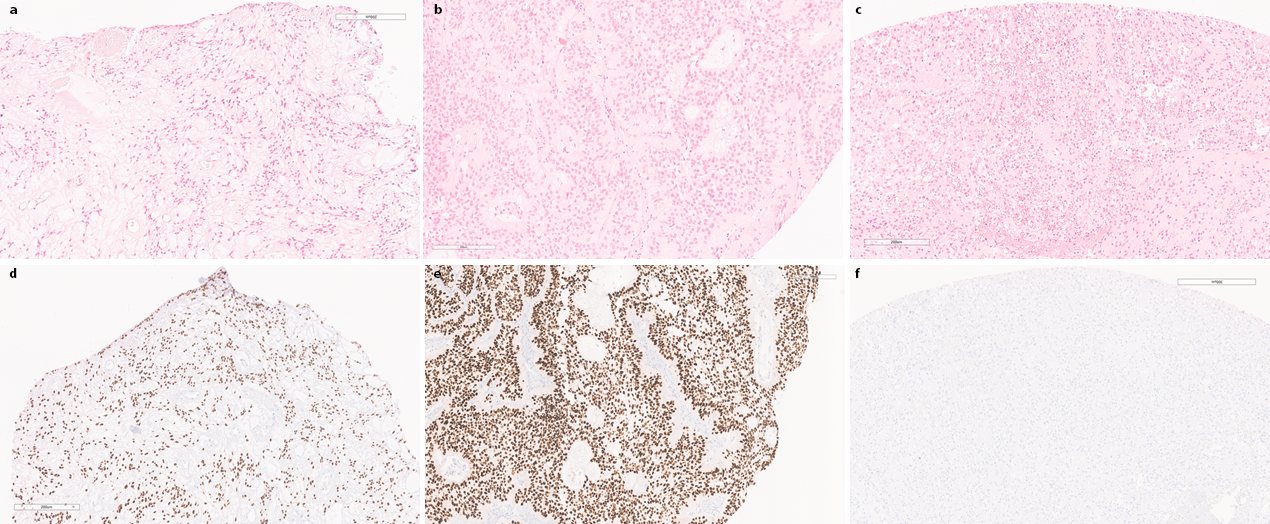


**Suppl Fig. 3** Pairwise comparison of HE slides and corresponding HOXB13 immunohistochemistry of myxopapillary ependymomas (a and d), discrepant cases (b and e) and spinal ependymomas (c and f).


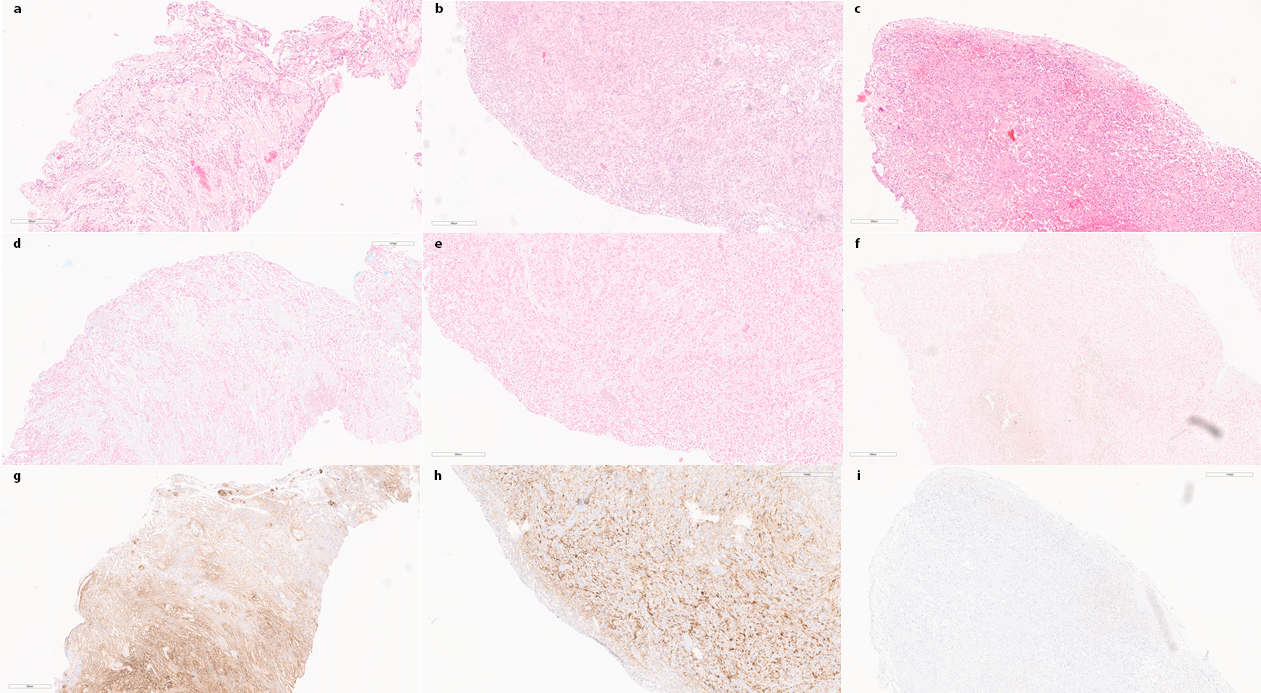


**Suppl Fig. 4** Pairwise comparison of HE slides, corresponding Alcian blue stain and VCAN immunohistochemistry of myxopapillary ependymomas (a, d and g), discrepant cases (b, e, h) and spinal ependymomas (c, f and i).


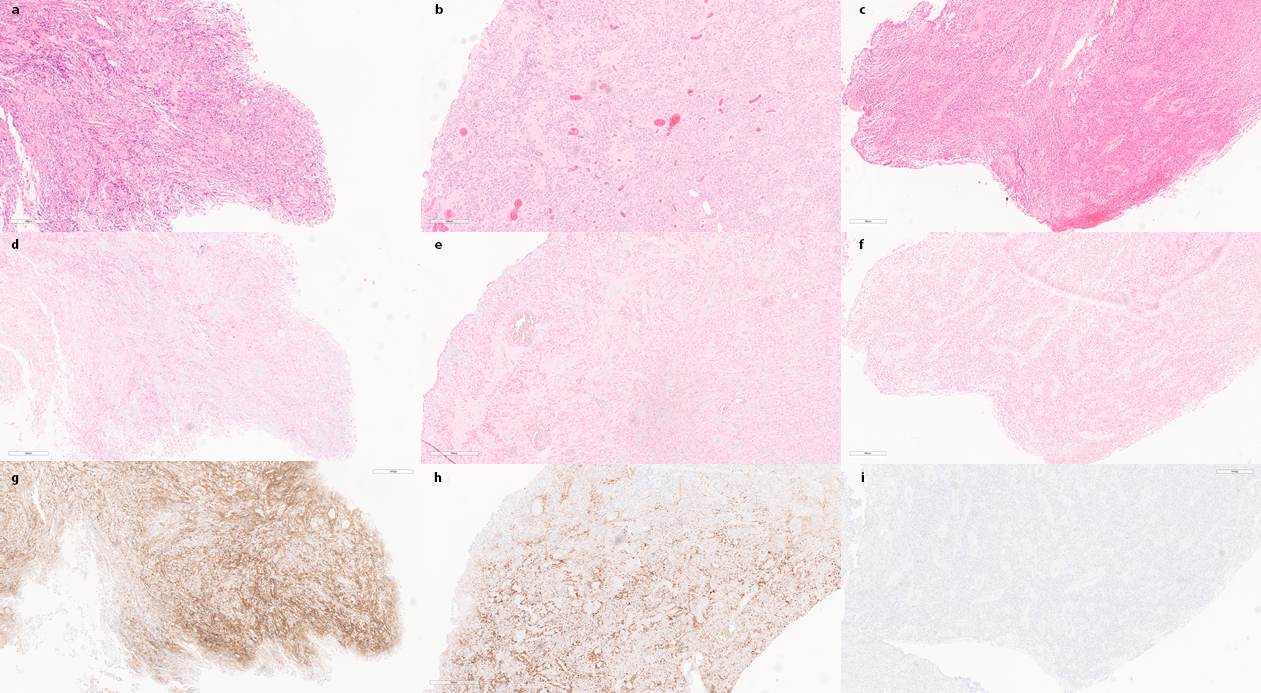


**Suppl Fig. 5** Pairwise comparison of HE slides, corresponding Alcian blue stain and VCAN immunohistochemistry of myxopapillary ependymomas (a, d and g), discrepant cases (b, e, h) and spinal ependymomas (c, f and i).


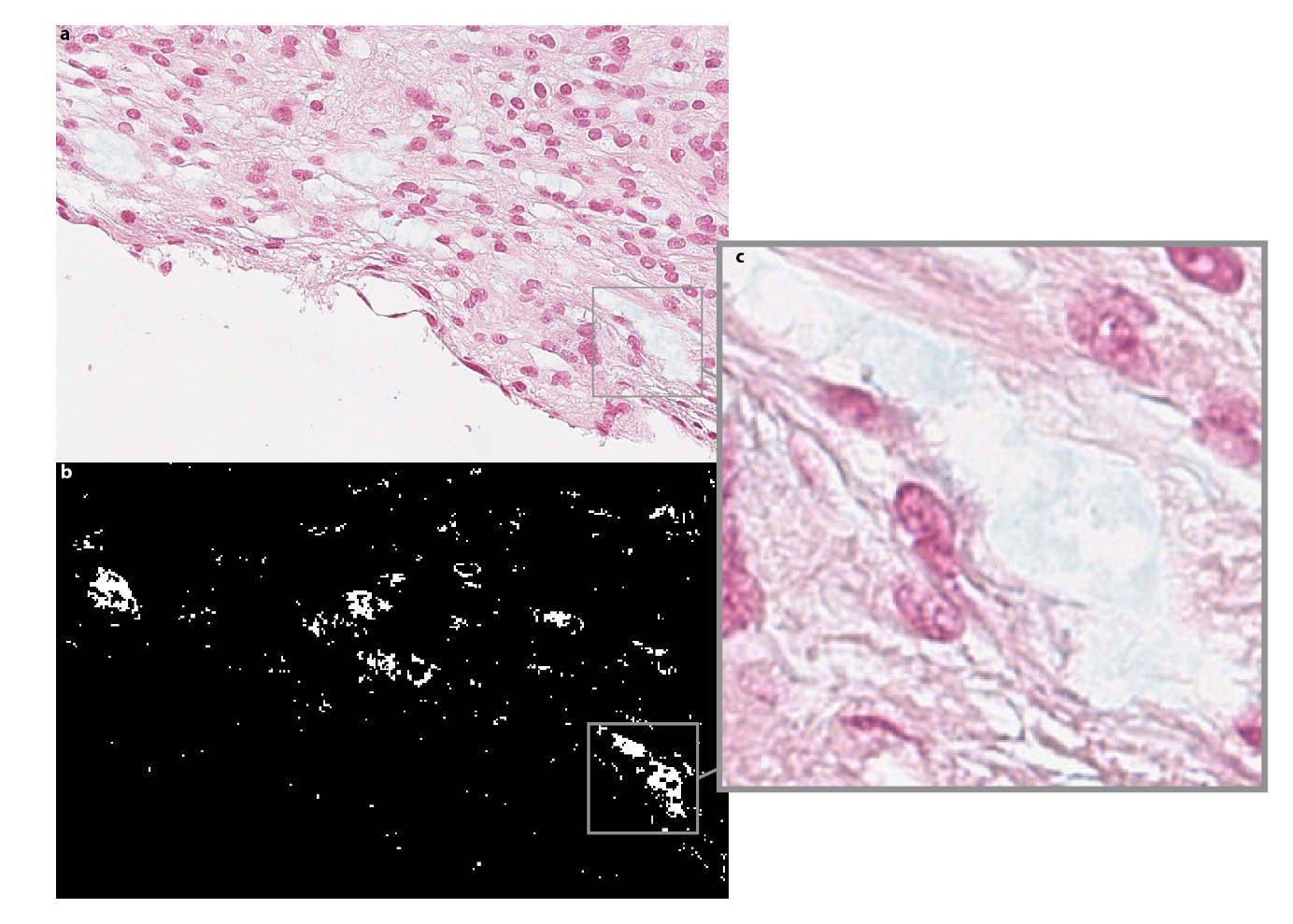


**Suppl Fig. 6** Alcian blue stain of a discrepant case (a) and mask output for the Alcian blue (b) indicating small and hard to detect myxoid foci (c) with the help of digital pathological tools.


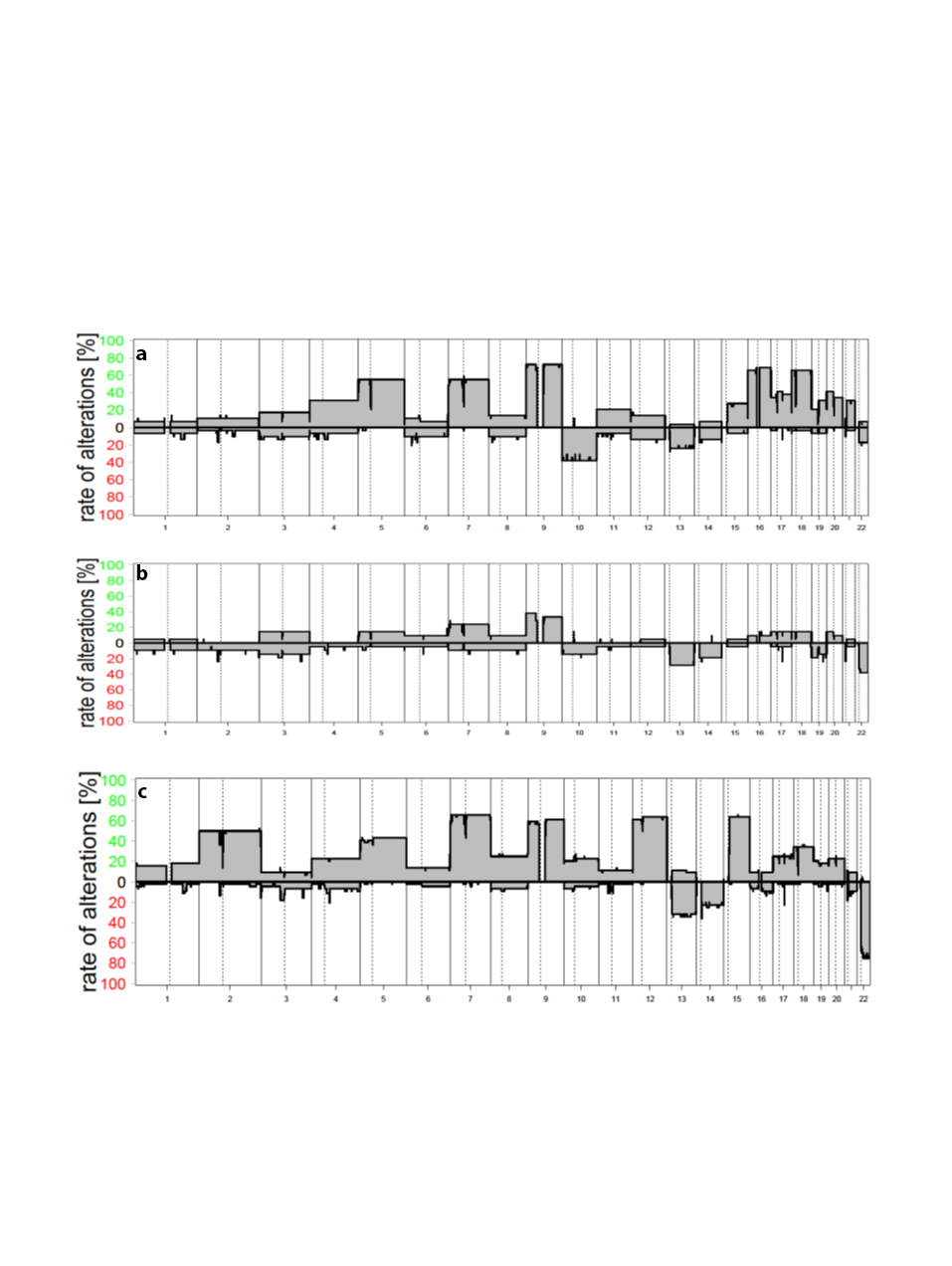


**Suppl Fig. 7** Summary of CNV plots of myxopapillary ependymomas (a), discrepant cases (b) and spinal ependymomas (c).

**REFERENCES**

1. Capper D, Jones DTW, Sill M, et al. (2018b) DNA methylation-based classification of central nervous system tumours. Nature 555:469–474. https://doi.org/10.1038/nature26000
2. Ritchie ME, Phipson B, Wu D, et al. (2015) limma powers differential expression analyses for RNA-sequencing and microarray studies. Nucleic Acids Research 43:e47. <https://doi.org/10.1093/nar/gkv007>
3. Hughes CS, Foehr S, Garfield DA, et al. (2014) Ultrasensitive proteome analysis using paramagnetic bead technology. Molecular Systems Biology 10:. https://doi.org/10.15252/msb.20145625
4. Demichev V, Messner CB, Vernardis SI, et al. (2019) DIA-NN: neural networks and interference correction enable deep proteome coverage in high throughput. Nature Methods 17:41–44. https://doi.org/10.1038/s41592-019-0638-x
5. Čuklina J, Lee CH, Williams EG, et al. (2021) Diagnostics and correction of batch effects in large‐scale proteomic studies: a tutorial. Molecular Systems Biology 17:. https://doi.org/10.15252/msb.202110240
6. Gu Z, Eils R, Schlesner M (2016) Complex heatmaps reveal patterns and correlations in multidimensional genomic data. Bioinformatics 32:2847–2849. https://doi.org/10.1093/bioinformatics/btw313
7. Benjamini Y, Hochberg Y (1995) Controlling the false discovery Rate: A practical and powerful approach to multiple testing. Journal of the Royal Statistical Society Series B (Statistical Methodology) 57:289–300. <https://doi.org/10.1111/j.2517-6161.1995.tb02031.x>
